# The CO-Regulation Database (CORD): A Tool to Identify Coordinately Expressed Genes

**DOI:** 10.1371/journal.pone.0090408

**Published:** 2014-03-05

**Authors:** John P. Fahrenbach, Jorge Andrade, Elizabeth M. McNally

**Affiliations:** 1 Department of Medicine, The University of Chicago, Chicago, Illinois, United States of America; 2 Center for Research Informatics, The University of Chicago, Chicago, Illinois, United States of America; 3 Department of Human Genetics, The University of Chicago, Chicago, Illinois, United States of America; Centro de Investigacion Principe Felipe, Spain

## Abstract

**Background:**

Meta-analysis of gene expression array databases has the potential to reveal information about gene function. The identification of gene-gene interactions may be inferred from gene expression information but such meta-analysis is often limited to a single microarray platform. To address this limitation, we developed a gene-centered approach to analyze differential expression across thousands of gene expression experiments and created the CO-Regulation Database (CORD) to determine which genes are correlated with a queried gene.

**Results:**

Using the GEO and ArrayExpress database, we analyzed over 120,000 group by group experiments from gene microarrays to determine the correlating genes for over 30,000 different genes or hypothesized genes. CORD output data is presented for sample queries with focus on genes with well-known interaction networks including p16 (*CDKN2A*), vimentin (*VIM)*, MyoD (*MYOD1*). *CDKN2A*, *VIM*, and *MYOD1* all displayed gene correlations consistent with known interacting genes.

**Conclusions:**

We developed a facile, web-enabled program to determine gene-gene correlations across different gene expression microarray platforms. Using well-characterized genes, we illustrate how CORD's identification of co-expressed genes contributes to a better understanding a gene's potential function. The website is found at http://cord-db.org.

## Background

More than one million gene microarray expression samples are available in the public domain through the Gene Expression Omnibus (GEO) and ArrayExpress databases. Such array databases include information regarding changes in gene expression that vary not only with the tissue or cell type being addressed but also specific to the conditions under which it was examined. Tools are available to group or organize gene expression results, most of which rely on comparing results across a limited number of samples and/or organizing the differentially expressed genes into biological groups to help interpret the findings. Such network analysis is broadly useful to determine how an experimental condition is acting.

“Added-value databases” process and analyze gene expression data to provide meta-analyses to extend what can be learned from the primary data. Most typically, such added-value databases utilize web-based approaches to make these additional analyses readily available [1]. Such tools permit *gene-centered queries*, e.g. identifying the experimental conditions under which a gene may be differentially expressed, or *experiment-centered queries,* in which the results are compared across experimental conditions [2], [3], [4]. The gene-centered databases generally focus on either identifying in which experiments a gene is differentially regulated or determining gene-gene correlation using expression value across a defined microarray platform. Several gene-centered added-value databases are freely assessable including GeneChaser and the Gene Expression Atlas [5], [6]. Both these tools allow the operator to assess in which experiments a gene may be differentially expressed. COXPRESdb and the Multi-experiment Matrix are two additional, freely accessible applications that can determine gene-gene associations [7], [8]. Commercially available value-added databases such as Nextbio and Genevestigator each have similar capabilities but offer additional analytics and generally more in-depth curation [9], [10].

Missing from currently available added-value database is an approach to allow for gene specific queries across different microarray platforms to determine what genes are coordinately expressed with a given gene. Such information is highly informative since its highlights potential gene-gene interactions. Current gene-gene associations are often determined by finding the correlation between the expression values across every microarray sample for each gene pair. Although these approaches provide valid information, the usefulness in determining gene-gene associations is limited since genes can be correlated even though they are not differentially regulated. Such analyses will include microarray experiments where the gene of interest is not differentially regulated which may diminish correlation values and, importantly, the analysis is limited to only one microarray platform. We now devised the CO-Regulation Database (CORD) database, an alternative approach that allows an investigator to query and identify in which gene expression datasets a given gene is differentially expressed, and then secondly to identify what genes are coordinately expressed with the gene of interest. Gene-gene correlations are determined using the fold-change expression for each gene pair using these experiments instead of raw expression values. To demonstrate the functionality of CORD, we queried genes with known expression partners. We also created a web application to make CORD readily available (http://cord-db.org).

## Methods

### Microarray Database Curation and Analyses

**Figure 1:**
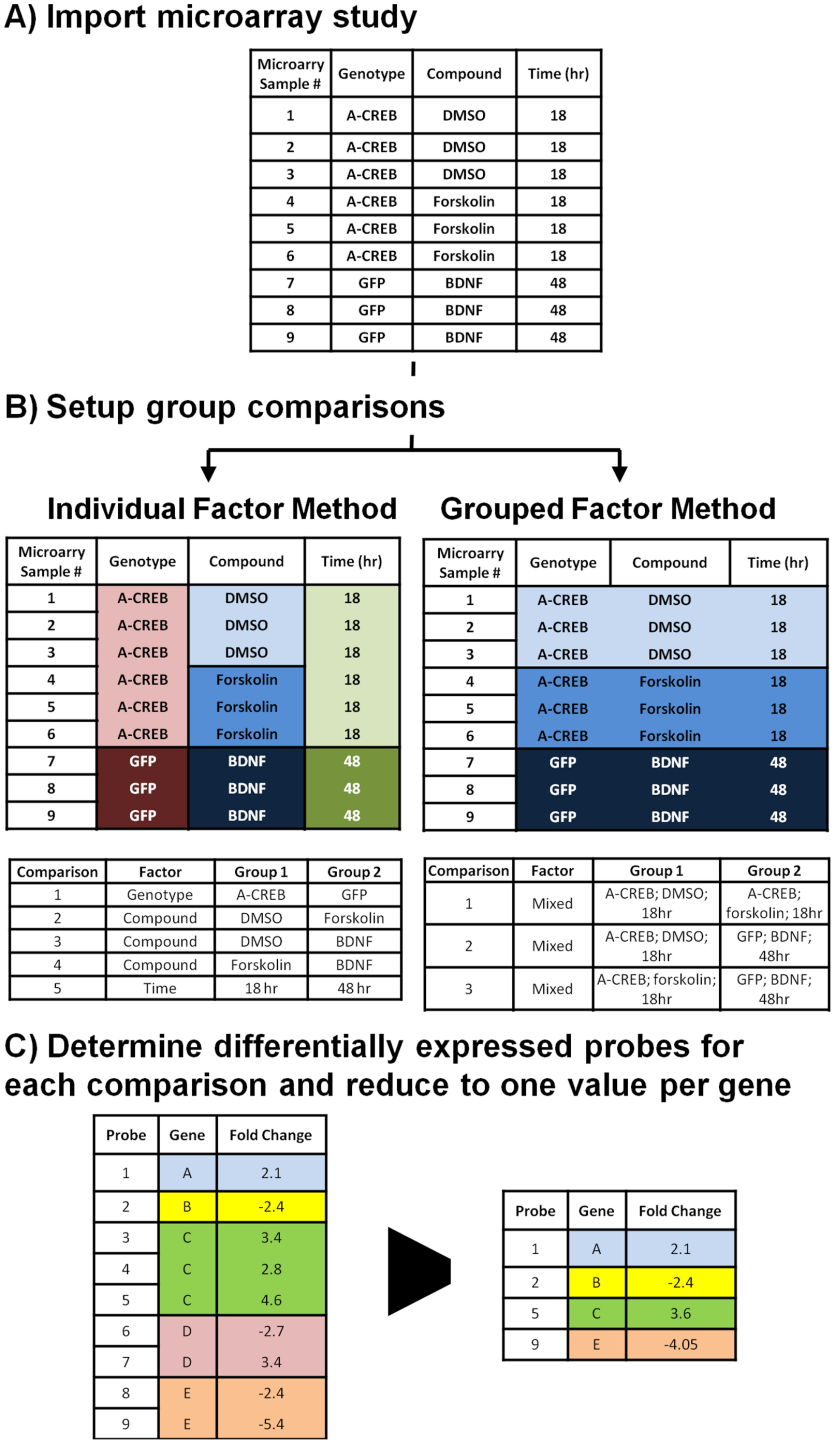
Construction of CORD database using a gene-centric approach. **A**) A part of the microarray study E-MEXP-3167 from the ArrayExpress database. **B**) The samples were grouped together by either the Individual or Grouped Factor Method and all the groups were compared to one another. **C**) The differentially genes for each comparison was determined. Genes with multiple probes were reduced to one entry by averaging the fold change for the multiple probes. If the multiple probes for a gene were differentially regulated in the opposite direction, the gene was removed from the list of differentially expressed genes.

9490 microarray datasets from *Homo sapiens*, *Mus musculus*, and *Rattus novegicus* experiments were downloaded and analyzed from the Gene Expression Omnibus (GEO) datasets and Affymetrix datasets in the Array Express database. From the GEO database, only datasets manually curated by the GEO staff were used. From the ArrayExpress database, we only analyzed datasets with factors defined according to MIAME standards. Because these datasets were curated by respective staffs, the factors could be reliably extracted from the experimental descriptions and used for setting up group by group comparison for differential gene expression analysis. For example, E-MEXP-3167 in the ArrayExpress database is a study on activity-driven neuronal gene expression in mice with three factors: genotype, compound, and time. In a subset of E-MEXP-3167, the genotype, compound, and time factors have 2, 3, and 2 variables respectively (Figure 1A). To generate microarray groups for differential gene expression analysis, microarray samples were group by individual or grouped factors. Using a part of E-MEXP-3167 as an example, all samples were grouped by only the variables in one factor (“Individual Factor Method’”) irrespective of the other factors that resulted in five comparisons (Figure 1A). To create subsets using the “Grouped Factor Method”, microarray samples with the same variables across all the factors were grouped resulting in three comparisons (Figure 1B). Using the Individual Factor Method, a total of 67169 comparisons were made. Using the “Grouped Variable Method” resulted in 64537 comparisons. Normalized data were obtained for all GEO datasets. The raw data from Affymetrix microarrays in the ArrayExpress database were normalized using the robust multi-array average (rma) method. Differentially expressed transcripts were determined by the empirical Bayes method in “limma” package in R/Bioconductor [11]. All p-values were adjusted using Benjamini and Hochberg's method to control the false discovery rate and a p value <0.01 was considered significant [12]. An example R script used for automated analysis is provided (Code S1). All orthologous human, mouse, and rat genes were linked to the human gene symbol to ensure gene symbol uniformity across species. Orthologous gene information was retrieved using the Mouse Genome Informatics (MGI) ortholog database (http://www.informatics.jax.org/orthology.shtml).

Microarrays are often designed with multiple probesets to a single transcript and some probes can distinguish multiple transcripts from a single gene locus. To simplify meta-analysis across different microarray platforms, the probesets to each gene locus were condensed to the average fold change for all the differentially expressed probesets (Figure 1C). If probesets to the same gene locus were found to differentially express in opposite directions, all probes to that gene locus were removed from the list of differentially expressed genes. For instance, the study E-TABM-877 from the ArrayExpress database was done with the Affymetrix GeneChip Mouse Genome 430 2.0. Probes 1440770_at and 1437122_at show a fold change of -1.95 and 1.09 respectively when comparing the adipose to muscle tissue. Since both probes target the same Refseq transcript, NM_009741, for the *Bcl2* gene, *Bcl2* was omitted as a gene that is differentially regulated in this comparison since the probe results gave opposite results. This situation had an occurrence rate of 2.74% among every analyzed gene. The 35654 and 42648 comparisons derived from the Individual and Grouped Factor methods had at least one differentially expressed gene. Using an adjusted p-value of 0.05, the number of differentially expressed genes averaged 1348±17 and 1014±13 using the Individual or Grouped Factor methods, respectively.

### Co-regulated gene ranking algorithms

To identify genes were co-expressed with a given target gene, all the experiments in which the target gene was differentially regulated were collected. Following that the Pearson correlation of log2 fold change of the target gene to every gene that was differentially regulated in those experiments was determined. Generating a final list of correlated genes is challenged by the lack of an agreed upon consensus threshold for p-value or correlation coefficient significance. Weak, although statistically significant, correlations often occur between variables when working with large datasets that are not informative. Illustrating this point was the observation that random gene lists were shown to correlate with breast cancer outcome [13]. Therefore, to refine the list of correlated genes, we choose to measure the robustness of each gene's correlation to the target gene by comparing the gene lists using the Individual and Grouped Variable methods. To estimate how the co-regulated gene lists differed between each method, the co-regulated gene lists for 1000 random target genes were generated using both methods. First all the co-regulated genes were rank ordered by its Pearson Correlation value for each method. Next, the percentage of common genes between the two gene lists (% overlap) was determined using the top 10 to 1000 genes in both lists (Equation 1). The co-regulated gene lists from the Individual and Grouped Factor method are represented by variables A and B respectively.

**Figure 2:**
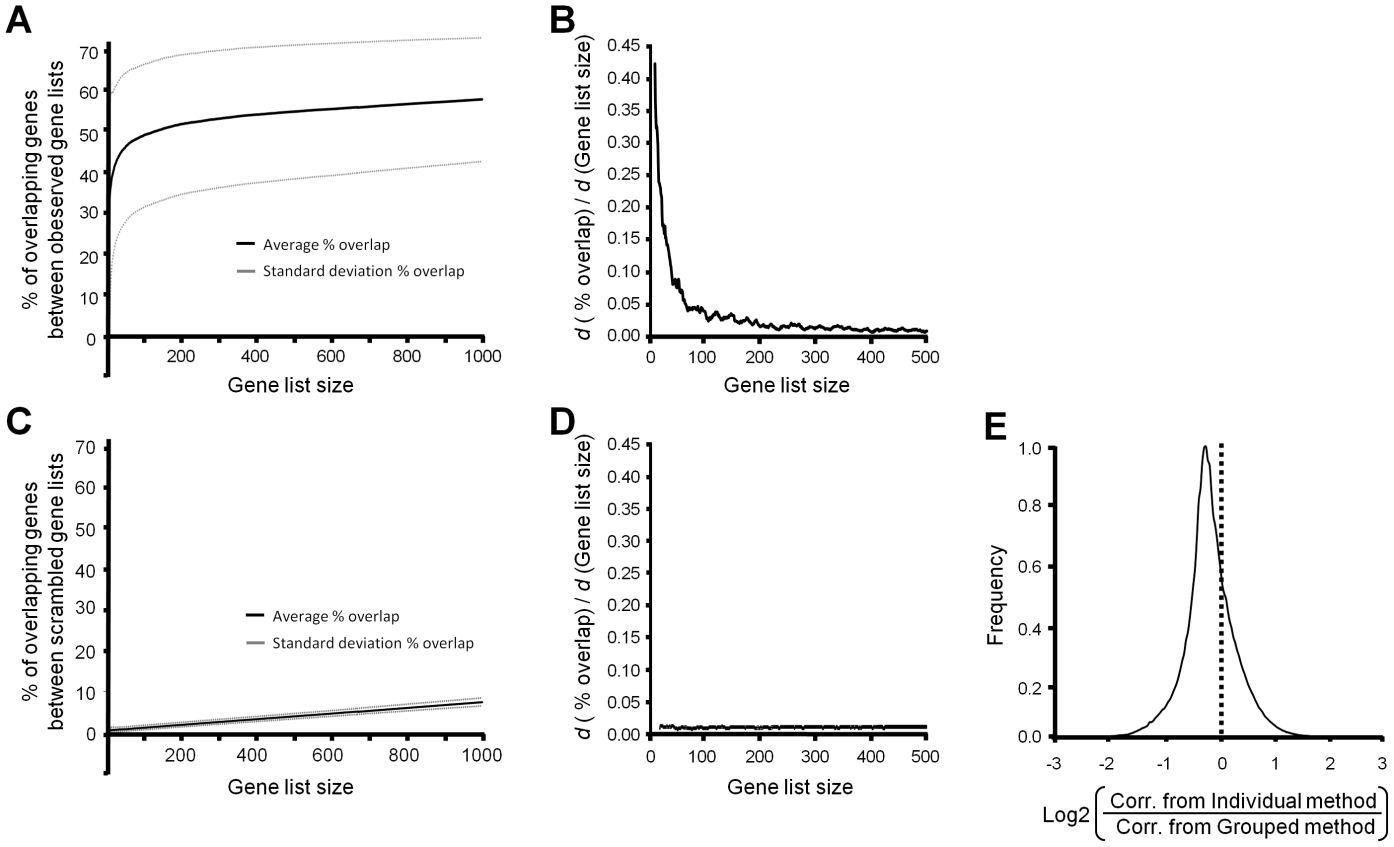
Determination of co-regulated genes. **A**) The list of co-regulated genes was determined for each gene using the Individual and Grouped Factor Method. The two gene lists were then compared to one another by determining the % overlap (similarity) of the lists for the top 10 to top 1000 most correlated genes. The % overlap reached a plateau at 47%. **B**) The first derivative of the % overlap vs. the gene list size shows that on average, after comparing the top 400 genes the lists are no longer similar. **C, D**) This analysis was repeated for randomly generated gene lists and showed no change in the rate of % overlap vs. gene list size. **E**) To determine how using the Individual or Grouped Factor method effected gene-gene correlation co-efficients, we analyzed the ratio of the correlation co-efficient for each gene-gene pair. A histogram of this data shows that on average, the Grouped Factor method yielded higher correlation co-efficients.

Equation 1:
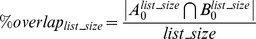



Equation 2:
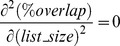



The % overlap increased asymptotically followed by a linear increase in relationship to gene list size (Figure 2A). For instance, when examining the top 50 genes in the co-regulated gene list from each method, the gene lists had 39.3% of genes in common. When examining the top 400 genes in the co-regulated gene list from each method, the gene lists had 52.1% of genes in common. A random overlap between two gene lists will always occur since the number of genes is finite. To quantitate the probability of random association, the ranked ordered gene lists generated from the Individual and Grouped Factor method for each tested gene were scrambled and the % overlap vs. gene list size relationship was similarly determined (Figure 2C). The % overlap displayed a small linear increase vs. gene list size similar to the second phase of Figure 2A. Taking the first derivative of the % overlap of the actual vs. random gene lists (Figure 2B, D), the linear phase of the % overlap in the actual gene list is most likely attributed to random overlap and is non-informative. The informative gene list size was taken as the gene list size where the % overlap curve becomes linear, similar to the random gene lists, or where the second derivate reaches 0 which was ∼400. Therefore the final informative correlated gene list was defined as the overlapping genes from top 400 genes from the gene lists generated by Individual and Grouped Variable methods.

To determine the final Pearson correlation value for each gene, the strength of correlations for the Individual and Grouped Variable methods was determined from the histogram of the log2 ratio of the Individual vs. Grouped Factor method Pearson correlation for each overlapping gene. If each method shows equal correlation for each gene, then a normalize distribution centered at 0 would be expected. However, since the plot shows a normal distribution centered at −0.42, the Grouped Variable method on average found more robust correlations than the Individual Variable method (Figure 2E). Therefore the overlapping genes were rank ordered by the Pearson correlation value from the Grouped Variable method.

## Results and Discussion

To demonstrate the functionality of CORD, we examined whether CORD could document expected gene partners for three genes with known gene networks and function. We applied CORD to p16 (*CDKN2A*), a cell cycle protein, vimentin (*VIM*), a cytoskeletal protein important for epithelial to mesenchymal transition (EMT), and the transcription factor, myogenic differentiation 1 (*MYOD1*), a master regulator of myogenesis. The CORD output files for *CDKN2*A, *VIM*, and *MYOD1* are included as File S1, File S2, and File S3.

**Figure 3:**
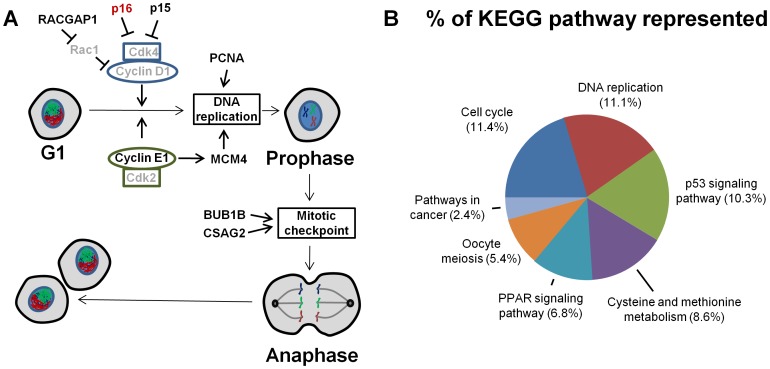
CORD results for *CDKN2A*. **A**) *CDKN2A* encoding p16 plays a significant role in the cell cycle by regulating the initiation of DNA replication. A simplified diagram shows select genes that play a major role in the cell cycle. CORD identifies many genes known to play major roles in the cell cycle by determining genes co-regulated with *CDKN2A* (bolded text.) **B**) The *CDKN2A*-correlated genes were over representative for several KEGG pathways in cancer and the cell cycle including “DNA replication”, “p53 signaling”, and “cell cycle.”

p16 encoded by the *CDKN2*A gene plays a central role in the regulation of cell cycle events [14]. Generally, cellular proliferation is inhibited by p16, which prevents the G_1_ to S transition by inhibiting cyclin-dependent kinase 4 (*CDK4*). Promoter methylation, homozygous deletion, single nucleotide polymorphisms, and up/down regulation of *CDKN2A* are prevalent in a wide array of cancers such as oropharyngeal, gastrointestinal, hepatic, skin, and ovarian cancers and emphasize its role as a tumor-suppressor gene [15], [16], [17], [18]. Using the factor method of CORD, *CDKN2A* was differentially regulated in 669 experiments using the default settings. CDKN2A role in cancer was evident by its inclusion of several cancer related experiments such as cervical (GDS3233), melanoma (GDS1375; GDS3012), prostate (GDS1439), and lymphatic (E-GEOD-29986; GDS 3516) cancers. *CDKN2A* displayed a maximal log2 fold change of 9.8 with an average value of 2.7±1.6. CORD of genes expressed with a similar pattern to *CDKN2A* supported its role in cell cycle regulation, as 18 of the top 20 co-expressed genes are genes known to be important for cell cycle or cancer (Figure 3A). Genes encoding p15 (CDKN2B) and cyclin E1 (*CCNE1*), and Rac GTPase activating protein 1 (*RACGAP1*) also regulate the G1-S transition and were the first, second, and eight most correlated genes of co-expressed genes with *CDKN2A*. Genes playing a central role in DNA replication including proliferating cell nuclear antigen (*PCNA*) and minichromosome maintenance complex component 4 (*MCM4*), and the mitotic check point, CSAG family, member 2 (*CSAG2*) and budding uninhibited by benzimidazoles 1 homolog beta (*BUB1B*), were also highly co-regulated with *CDKN2A*. Several other genes in the top 20 were also related to cancer. For example, methylthioadenosine phosphorylase (*MTAP*), which is only separated from *CDKN2A* by 100kB, is often co-deleted with CDKN2A in several cancers [19]. Melanoma antigen family C1 (*MAGEC1*) was also correlated with *CDKN2A* and is associated with several cancers [20], [21]. The KEGG pathways enriched in the list of all co-regulated genes were also examined (Figure 3B). As expected, genes involved in the cell cycle pathway (KEGG entry: hsa04110) were the most significant and followed by p53 signaling (KEGG entry: hsa04115), DNA replication (KEGG entryhsa03030), Oocyte meiosis (KEGG entry: hsa04114), PPAR signaling pathway (KEGG entry: hsa03320), pathways in cancer (KEGG entry: hsa05200), and cysteine and methionine metabolism (KEGG entry: hsa00270). The identification of known *CDKN2A*-interacting genes and the overrepresentation of cell cycle and cancer pathways demonstrated that CORD correctly identified the CDKN2A gene network.

**Figure 4:**
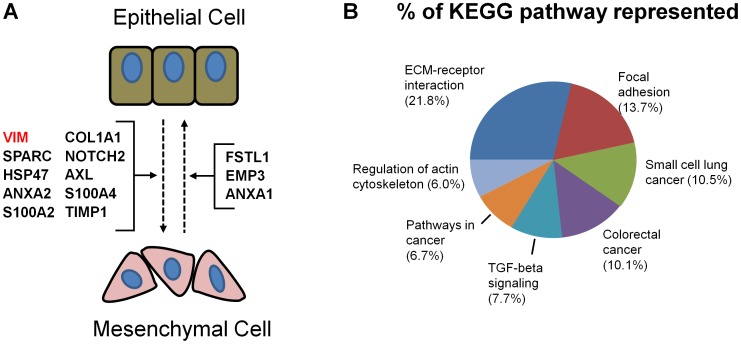
CORD results for *VIM*. **A**) The epithelial-to-mesenchymal (EMT) and mesenchymal-to-epithelial transitions are important oncogenic pathways where vimentin (*VIM*) plays a central role. Twelve of the top 20 most correlated *VIM* genes affect the EMT transition. **B**) The EMT transitions depend heavily on cell adhesion. The *VIM*-correlated genes were over representative for several KEGG pathways in cell adhesion and cancer pathways such as “ECM-receptor interaction”, “Focal adhesion”, and “Pathways in cancer.”

The epithelial-mesenchymal transition (EMT) is a reversible process where epithelial cells loss cell polarity and gain migratory capacity. A major part of the EMT process is the reorganization of the cytoskeletal especially focal adhesions. EMT occurs during development, wound healing, fibrosis, and cancer metastasis [22]. Vimentin is an intermediate filament protein that is critical for EMT through its role in focal adhesion formation and cell motility [23], [24]. Vimentin is also implied in tumor growth and metastasis by upregulating AXL receptor tyrosine kinase (AXL) [24], [25]. In the CORD database, vimentin was differentially expressed in 1531 different experiments. The previously identified role of vimentin in cancer was evident due to its upregulation in several cancer experiments including colon (E-GEOD-34053; GDS756; GDS2609; GDS2947), cervical (GDS3233), and breast (E-GEOD-22865) cancers. Of the top 20 most correlated genes with vimentin, 13 are known to play a direct role in EMT (Figure 4A; Table 1). Figure 4B shows the top 7 KEGG pathways that were enriched in the 239 VIM correlated genes. Several functional pathways involved in EMT such as ‘ECM-receptor interaction’, ‘Focal adhesion’, ‘regulation of the actin cytoskeleton’, and ‘TGFβ signaling’ were overrepresented. Additionally, the identification of the colorectal cancer pathway was expected since vimentin is a recently identified therapeutic target [26], [27]. The identification of known EMT interacting genes with vimentin and the overrepresentation of vimentin associated genes in EMT processes further demonstrates the utility of CORD.

**Table 1:** Top 20 Genes Co-expressed with vimentin (*VIM*) identified by CORD.

Gene symbol	Description	EMT	References
ANXA1	annexin A1	x	[34]
S100A6	S100 calcium binding protein A6	x	[35]
ANXA2	annexin A2	x	[36]
CAPG	capping protein (actin filament), gelsolin-like	x	[37]
LGALS1	lectin, galactoside-binding, soluble, 1		
SPARC	secreted protein, acidic, cysteine-rich (osteonectin)	x	[38]
S100A4	S100 calcium binding protein A4	x	[39]
EMP3	epithelial membrane protein 3	x	[40]
AXL	AXL receptor tyrosine kinase	x	[41]
SERPINH1	serpin peptidase inhibitor, clade H, member 1		
TIMP1	TIMP metallopeptidase inhibitor 1	x	[42]
COL1A1	collagen, type I, alpha 1	x	
	calponin 2		
LGALS3	lectin, galactoside-binding, soluble, 3		
FSTL1	follistatin-like 1	x	[43]
GPX8	glutathione peroxidase 8 (putative)		
RBMS1	RNA binding motif, single stranded interacting protein 1		
COL5A2	collagen, type V, alpha 2		
NOTCH2	notch 2	x	
CMTM3	CKLF-like MARVEL transmembrane domain containing 3		

**Figure 5:**
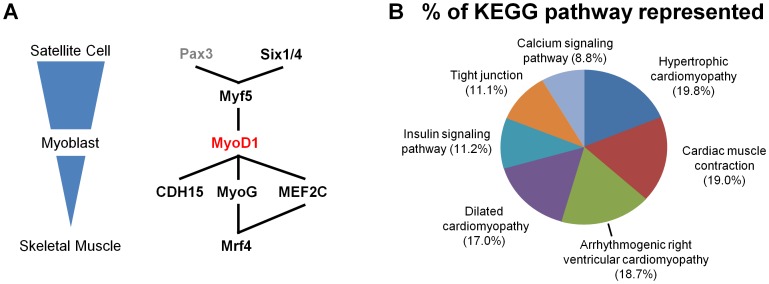
CORD results for *MYOD1*. **A**) The differentiation of muscle stem cells (satellite cells) to myoblasts and ultimately to skeletal muscle is under the control of muscle regulatory factors including the transcription factor MyoD. CORD output for *MYOD1* demonstrates co- expression of other muscle regulatory factors like myogenin (*MYOG*) and many genes implicated in muscle differentiation. **B**) The MyoD1 correlated genes were over representative for several KEGG pathways relating to muscle such as “Cardiac muscle contraction” and “Dilated cardiomyopathy.”

CORD analysis was also conducted using *MYOD1*, which plays a coordinated role in myogenic specification [28], [29], [30], [31], [32]. The myogenesis gene network, where *MYOD1* plays a central role, displays a complex temporal and spatial morphology [30] (Figure 5A). Using the Factor method, CORD extracted 397 comparisons where *MYOD1* was differentially expressed. *MYOD1* displayed a large variation of differential expression ranging 2.0 fold (set at default) to 99.0 fold (average fold change 7.7±8.9) (Figure 3A). The overexpression of *MYOD1* in MyoD1- overexpressing fibroblasts vs. normal fibroblasts (GEO: GDS2854) validated the specificity of the batch data retrieval and analysis used in CORD. As anticipated, developmental and damage-induced myogenesis experiments showed an increase in *MYOD1* expression (GDS586; E-GEOD-16992; GDS2158; GDS2158). Since MyoD1 is a transcription factor, we compared the *MYOD1* correlated genes from CORD to UCSC MyoD1 Chip-Seq dataset from muscle cells. MyoD1 was shown to bind to 63% of the promoters in the *MYOD1* gene list. Several of the genes that play a prominent role in myogenesis were strongly correlated with *MYOD1*. Myogenin (*MYOG*), myogenic factor 5 (*MYF5*), and myogenic factor 6 (*MYF6*; herculin) are other transcription factor critical for myogenesis [33] and were on the list of correlated genes demonstrating the utility of CORD.

## Conclusion

The large collection of gene expression microarrays in public data repositories offers a wealth of information for exploring and generating novel hypotheses. Using a gene-centered approach, we developed a method to extract experiments where a gene is differentially regulated and then determine which genes are similarity co-regulated. Both the GEO and ArrayExpress databases allow an investigator to query in which experiments a gene of interest is expressed. We now amplified this capacity in order to identify co-expressed genes sampling multiple gene expression datasets simultaneously. By sampling multiple gene sets, the list of co-expressed genes can inform significantly about the biological function of a given gene. The gene lists generated from CORD are influenced by the expected variables that contribute to co-expression such as tissue specificity and shared biological roles. It is for this reason that these results can prove highly useful when analyzing a gene whose function is not well known. Furthermore, the list of co-expressed genes may provide valuable information about previously unappreciated biological connections for known genes.

There are a number of limitations that derive from querying thousands of datasets from the GEO and ArrayExpress databases simultaneously. For example, different microarray platforms often utilize different probesets that may target different isoforms of the same gene. To address this issue, we condensed each list of differentially regulated genes for each experiment down to one entry per gene locus. Although we lose the ability to probe different gene isoforms, we gain the ability to use multiple microarray platforms, greatly increasing sample sizes. This capacity could be adapted in future releases in order to more carefully examine gene splicing changes. As RNAseq analysis and databases develop further, this capacity will prove more relevant.

In summary, CORD is a meta-analysis tool that queries gene expression databases in the public domain to identify lists of coordinately expressed genes. This tool will provide useful information that can then be combined with other tools such as those directed at gene ontology to provide information about the potential biological function of genes whose function is not well known. Knowledge of coordinately expressed genes can also point towards previously unappreciated roles for known genes. CORD is available as a freely available web application for wide use. By allowing open access to CORD, it is anticipated to aid in finding novel functions and correlated partner genes of a target gene

### Availability and Requirements

Cord is available at http://cord-db.org with a web interface for ease of use. To retrieve results for a gene of interest, enter the gene name into the ‘Target Gene(s)’ field. Multiple genes separate by a space character may also be entered and CORD will analyze only those experiments where entered genes were differentially regulated in the same manner. For instance, entering ‘MyoD1’ or ‘MyoD1 MyoG Myf7’ can be entered to look at a specific network of genes. Once a gene(s) of interest has been entered and filter set selected, CORD emails an Microsoft Excel file with 6 tabs. Tab 1 contains the information for each experiment where the gene(s) was differentially regulated. Tabs 2 and 3 contain genes with the most similar or dissimilar fold change differential expression in all the experiments listed in Tab 1. Tabs 4-6 delineates which KEGG pathways (Tab 4), COG (Tab 5), and tissues (Tab 6) are enriched in the gene list in Tab 2.

## Supporting Information

Code S1 A file (analyze_AE_dataset.R) containing the R source code for analyzing an ArrayExpress experiment using an Affymetrix array.
(R) Click here for additional data file.

File S1 An Excel file (CDKNA2.xls) containing the CORD results for *CDKNA2* using the following settings: human and mouse experiments used, p value of less than 0.01, fold change threshold of greater than 2, tissue comparisions included, and the minimum number of samples per experiment is 3.
(XLS)

File S2 An Excel file (VIM.xls)containing the CORD results for *VIM* using the following settings: human and mouse experiments used, p value of less than 0.01, fold change threshold of greater than 2, tissue comparisions included, and the minimum number of samples per experiment is 3.
(XLS)

File S3 An Excel file (MYOD1.xls)containing the CORD results for *MYOD1* using the following settings: human and mouse experiments used, p value of less than 0.01, fold change threshold of greater than 2, tissue comparisions included, and the minimum number of samples per experiment is 3.
(XLS)
